# MetaGenSense: A web-application for analysis and exploration of high throughput sequencing metagenomic data

**DOI:** 10.12688/f1000research.6139.3

**Published:** 2016-12-01

**Authors:** Damien Correia, Olivia Doppelt-Azeroual, Jean-Baptiste Denis, Mathias Vandenbogaert, Valérie Caro

**Affiliations:** 1 Pôle Génotypage des Pathogènes, Unité Environnement et Risques Infectieux, Institut Pasteur, F-75724, Paris, France; 2 Centre de Bioinformatique, Biostatistique et Biologie Intégrative (C3BI, USR 3756 Institut Pasteur et CNRS), Institut Pasteur, F-75724, Paris, France; 3 Groupe Exploitation et Infrastructure, Institut Pasteur, F-75724, Paris, France

**Keywords:** High Throughput Sequencing, Next-Generation Sequencing, Laboratory Information Management System, Galaxy, Django

## Abstract

The detection and characterization of emerging infectious agents has been a continuing public health concern. High Throughput Sequencing (HTS) or Next-Generation Sequencing (NGS) technologies have proven to be promising approaches for efficient and unbiased detection of pathogens in complex biological samples, providing access to comprehensive analyses. As NGS approaches typically yield millions of putatively representative reads per sample, efficient data management and visualization resources have become mandatory. Most usually, those resources are implemented through a dedicated Laboratory Information Management System (LIMS), solely to provide perspective regarding the available information.

We developed an easily deployable web-interface, facilitating management and bioinformatics analysis of metagenomics data-samples. It was engineered to run associated and dedicated Galaxy workflows for the detection and eventually classification of pathogens.

The web application allows easy interaction with existing Galaxy metagenomic workflows, facilitates the organization, exploration and aggregation of the most relevant sample-specific sequences among millions of genomic sequences, allowing them to determine their relative abundance, and associate them to the most closely related organism or pathogen.

The user-friendly Django-Based interface, associates the users’ input data and its metadata through a bio-IT provided set of resources (a Galaxy instance, and both sufficient storage and grid computing power). Galaxy is used to handle and analyze the user’s input data from loading, indexing, mapping, assembly and DB-searches. Interaction between our application and Galaxy is ensured by the BioBlend library, which gives API-based access to Galaxy’s main features. Metadata about samples, runs, as well as the workflow results are stored in the LIMS. For metagenomic classification and exploration purposes, we show, as a proof of concept, that integration of intuitive exploratory tools, like Krona for representation of taxonomic classification, can be achieved very easily. In the trend of Galaxy, the interface enables the sharing of scientific results to fellow team members.

## Introduction

### Background HTS & metagenomics

The detection and characterization of emerging infectious agents has been a continuing public health concern. High Throughput Sequencing (HTS; or Next-Generation Sequencing, NGS) technologies have proven to be efficient at providing access to comprehensive analyses and unbiased detection of pathogens in complex biological samples.

 Most large-scale genomic (re)sequencing projects involve both sequencing technology, computational analyses, and genotyping expertise. Current NGS platforms including Illumina, Ion Torrent/Life Technologies, Pacific Bioscience and Nanopore can generate reads of 100–10,000 bases long allowing better coverage of the genome at lower cost. However, these platforms also generate huge amounts of raw data.

Besides those sequence files, it has become important to also consider and store associated sample related metadata (collection date, location, …). In addition, NGS projects usually represent such a huge amount of relevant sample-specific sequences that efficient data management and visualization resources have become mandatory. Those challenges accompanying HTS technologies have raised the following fundamental questions: (1) How do we best manage the enormous amount of sequencing data? (2) What are the most appropriate choices among the available computational methods and analysis tools? The issue concerning the growing amount of data can be managed through a dedicated *Laboratory Information Management System* (LIMS), solely to organize and plan their analysis, and to provide perspective. The question regarding the lack of adapted intertwining among the wide spectrum of available tools was in part filled by workflow management systems, even though it still requires fairly advanced knowledge of the tools available at hand.

Indeed, today, hundreds of bioinformatics tools are available, each demanding specific parameterization ^1^. NGS data analysts have been designing workflows in order to automate complex processing pipelines, both possibly using existing codes, avoiding rewriting software, and supporting parallel, distributed computations. These features make the workflows relatively simple to construct, make them easily reusable, hence aid reproducibility. Modern workflow managers, such as Galaxy ^2– 4^, offer the possibility of sharing them with others. As such, Galaxy as a data analysis and workflow management system, provides biologists with a hands-on toolbox to build multi-step computational workflows for data-processing, quality control, and analytic results aggregation, while additionally ensuring analysis reproducibility. As a prerequisite to a system for composing pipelines for large-scale analyses, there is a need for an adapted and up-to-scale computational infrastructure capable of doing the processing and data storage.

We therefore developed MetaGenSense, a bioinformatics web-application framework whose principle purpose is to ease the scientists’ work in management of NGS-project related data and analysis results. MetaGenSense is built on three fundamental components, two of which are specific to the project: a dedicated LIMS and a Django-based web user-interface. The third component is Galaxy, which is the main bioinformatics workflow management system. In the following paragraphs, we describe the interface’s implementation and display how communication between the different parts takes place, in a smooth and user-friendly, managing web-user interface.

## Software tool - implementation

### MetaGenSense global description

MetaGenSense is a managing and analytical bioinformatics framework that is engineered to run dedicated Galaxy workflows for the detection and eventually classification of pathogens. It aims to facilitate large-scale genomic analysis for experts in sequencing among project partners. The web application was built and can be deployed in order to facilitate access to high throughput sequencing analysis tools, acting as an information resource for the project and interacting research partners. With its user-friendly interface, it was designed to take advantage of bio-IT provider resources (a local Galaxy instance, sufficient storage and grid computing power), for analysis of input data and its metadata. Also in MetaGenSense, a dedicated LIMS (postgreSQL-based) was implemented to ensure data coherence. The web interface design is based on the Django web framework ( http://www.djangoproject.com). Moreover, the communication with Galaxy is covered by the Bioblend library ^5^ which provides a high-level interface for interacting with the Galaxy application, promoting faster interaction, and facilitating reuse and sharing of scripts.

The use of the available Galaxy tools and workflows is automated and seamless with MetaGenSense. Galaxy, as a pipeline management software, lets you define workflows and “pushes” the data through that pipeline. The pipeline manager ensures that all the tools in the pipeline run successfully, generally spreading the workload over a computational cluster. For example, MetaGenSense is used at the Pasteur Institute to do the bulk of the data processing for a large number of HTS projects, and can be adapted to launch any set of instructions stitched together in a dedicated workflow available in the Galaxy workflow designer interface.

### A dedicated LIMS

**Figure S1.**
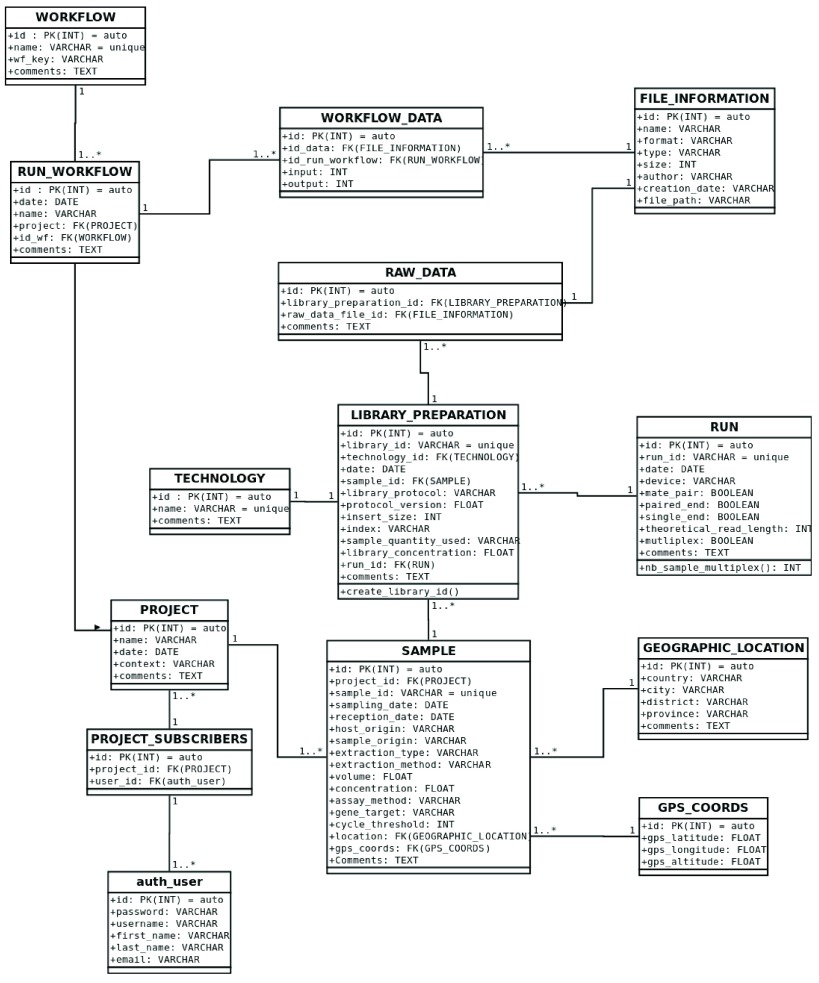
LIMS database schema.

A LIMS can be described as a software-based laboratory that offers a set of key features that support modern laboratory operations. Those systems have become mandatory to manage the quantity of metadata related to both raw data and their analysis results, obtained through bioinformatic tools. In MetaGenSense, the LIMS is based on a postgresql database. It was designed and structured with expert knowledge from biologists and bioinformaticians. Its main feature is that it was designed to store note-worthy and shareable information resulting from analysis, as well as the applied workflow-related parametrization. The database’s schema is available in the Supplementary Figure 1.

### An intuitive Django-based web user interface

Django is a high-level Python Web-framework. It provides rapid development, as well as clean pragmatic design, and serves as data management and displaying backbone for a large number of websites where interaction is important. Moreover, the python language ( https://www.python.org/) has become a reference programming language for a huge number of scientific applications.

MetaGenSense’s UI is divided in 4 modules: 1) User authentication management, 2) LIMS, 3) Workflow, 4) Analysis. Each has a specific function, and the task-partitioning has been designed to allow independent evolution of each part according to the user’s needs.

1. **User authentication management**: can be done either by communication with an LDAP user authentication database, or through a user management database.
2. **LIMS**: ensures the organization of the data according to the project at hand. The LIMS can be provided with sample metadata, which can be shared with selected project-members, and ensures sample traceability, which is an important component of any present-day core resource laboratory management system.
3. **Workflow**: manages **(a)** the connection with the Galaxy instance, **(b)** execution tracking (the Galaxy “user histories”) **(c)** the data from Galaxy “libraries” (the datasets), **(d)** association and import of data from a data-library to a Galaxy user-history instance. **(e)** Execution of the selected Galaxy workflow. This module handles data storage and links the samples to the selected workflow.
4. **Analysis**: A result file can be saved in order to be shared with other users involved in the project, or can be exported using the Galaxy export functionality, for download.

**Figure 1.**
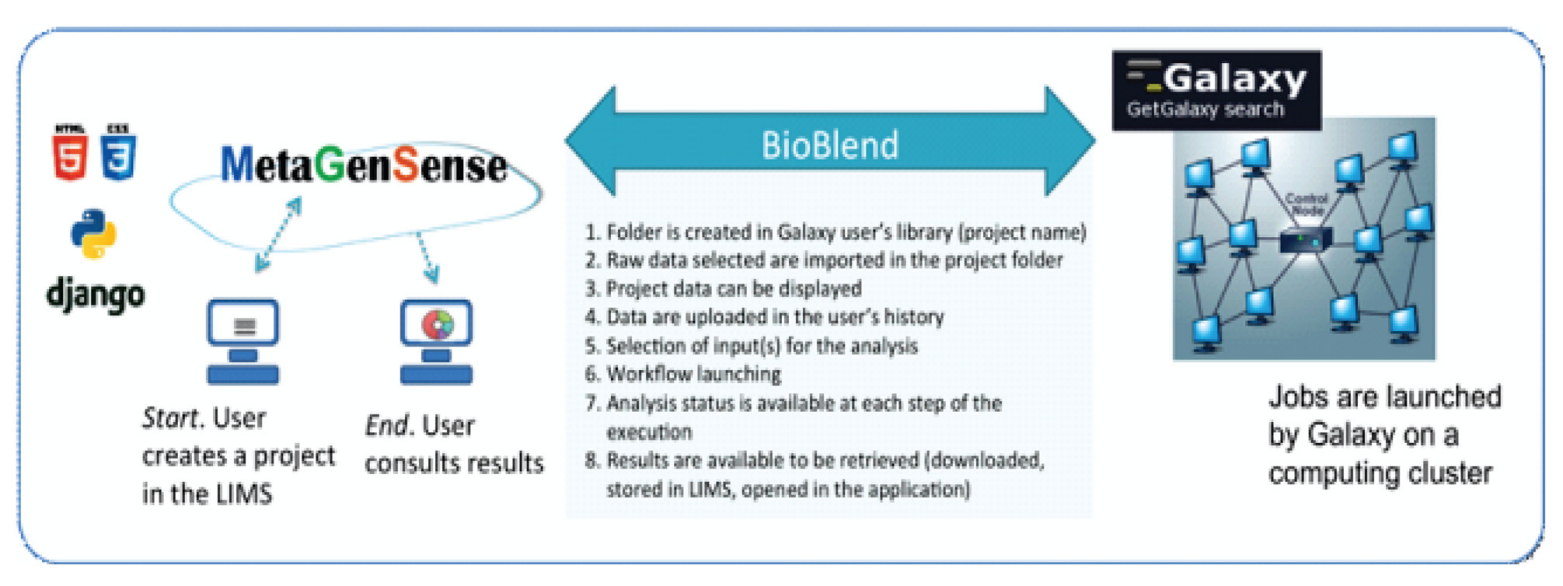
Communication details between MetaGenSense and Galaxy using Bioblend.

Communication between MetaGenSense and Galaxy is dealt systematically, using the BioBlend API ^5^, a highly dedicated and specialized python library, giving access to most Galaxy functionalities. For example, it is possible to fetch the Galaxy users, create the user’s data-libraries, … As a side-note, interaction with the BioBlend development team was necessary for fine-tuning specific core functionalities, leading to a concomitant finishing and perfection of the tools and accompanying API. Specifically, BioBlend functionalities are used to communicate with Galaxy, as described in Figure 1.

**Figure 2.**
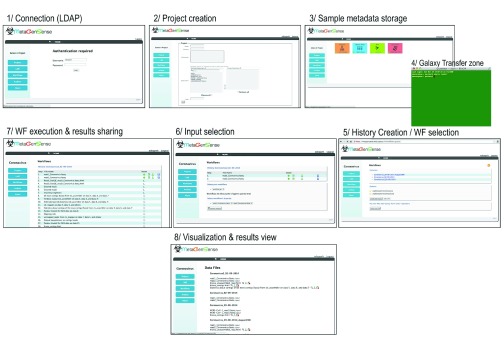
MetaGenSense functioning starting with user connection.

A use-case example of MetaGenSense typically involves the following steps (cf Figure 2).

• User Authentication with LDAP or any authentication system defined by admin sys.
• Creation of a new project, with a name, a context, a short description and (most importantly) the other persons involved in the project. Please note that to include a person, they need to have logged at least once on the application.
• Supplying the LIMS database with metadata including sample information as the library sequencing protocol, supplementary run details and the path to raw data files.
• At this point, the user needs to copy their input data files to their Galaxy transfer directory. MetaGenSense detects new files that are copied within the exchange Galaxy project directory. Those data files need to be copied manually. This solution was chosen such that the right files would be deposited deliberately in the right target Galaxy directory, with correct user/owner’s file permissions (typically on a UNIX file-system). In the exchange Galaxy directory, a subdirectory can be created, named after the project, and the raw data can be copied in that directory. This way, MetaGenSense detects the files that will be taken into account for use with Galaxy and analysed.
• In the MetaGenSense GUI, the “Workflows” button allows to import new files to analyse into Galaxy.
• Using a new Galaxy “history”, an appropriately selected workflow, and the set of workflow input data-files, an analysis can be launched. As usual in Galaxy, the workflow status can be monitored.
• Intermediate and final results can be exported for download (using the native Galaxy tools), or they can be saved in the LIMS *i.e.* be tagged as potentially interesting and shared with project members.
• Further exploration of workflow results can be done using, for example in a metagenomics data analysis context, Krona ^6^, that enables taxonomic information to be explored.

## Conclusions/Discussion

The technology evolution in molecular biology, especially in NGS, has moved biology into the big data era (consisting of handling data, computation requirements, efficient workflow design, and knowledge extraction). With this trend, the challenges faced by life scientists have been shifted from data acquisition to data management, processing, and knowledge extraction. While many studies have recognized the big data challenge, few systematically present approaches to tackle it. New findings in biological sciences usually come out of multi-step data pipelines (workflows). Galaxy is a workflow-management tool which can deal with big data. However, it is still necessary to globally optimize the data flow in an overall multi-step workflow in order to eliminate unnecessary data movement and redundant computation. On the other hand, data information traceability has become an inevitable requirement in a present-day laboratory setup, foreseeing that knowledge-embedded data and workflows are expected to be an integral part of future scientific publications.

We therefore, engineered MetaGenSense, a Django-based web interface which helps biologists, who in particular are unfamiliar with the design of Galaxy workflows, to quickly obtain analysis results from HTS sequencing projects. It uses Galaxy as a workflow management software and the BioBlend API to remotely manage data upload, workflow execution as well as analysis of results. MetaGenSense covers data processing up to presentation of data and results in a human-readable data format. Its main advantages encompass data handling through its incorporated LIMS, user and project handling in a cooperative context, it enables data sharing without compromising data confidentiality, it features automated workflow execution, resulting altogether in decreasing the data and analysis delivery time. MetaGenSense is available as open-source from GitHub, and can be deployed very easily. For testing, and for users to be able to evaluate MetaGenSense’s modularity, we added to our Github directory, a special release coupled with a virtual machine image containing, the tools needed in our metagenomic example workflow, a galaxy instance containing those tools as well as a prototyped workflow, mainly focused on metagenomic sample analysis. MetaGenSense is pre-configured on it and directly usable through a web browser. Once tested, it can be easily adapted to a variety of other NGS projects.

## Software availability

### Latest source code

https://github.com/pgp-pasteur-fr/MetaGenSense

### Source code as at the time of publication

https://github.com/pgp-pasteur-fr/MetaGenSense/releases/tag/1.0 (Official v1.0 MetaGenSense release coupled with a virtual machine image for testing)

### Archived source code as at the time of publication

DOI: 10.5281/zenodo.16510 ^7^

### License

GPLv2
