# Supplementary material for: MetaGenSense: A web-application for analysis and exploration of high throughput sequencing metagenomic data
Source: F1000Res. 2016 Dec 1;4:86. Originally published 2015 Apr 2. [Version 3] doi: 10.12688/f1000research.6139.3 (PMC5405795; doi:10.12688/f1000research.6139.3)
Supplement: Peer review history

# Peer review: Referee response for version 3

**DOI:** 10.5256/f1000research.11103.r18134

**Published:** 2016-12-01

**Authors:** Saskia Hiltemann

**Affiliations:** 1 Department of Bioinformatics, Erasmus University Medical Center, Rotterdam, Netherlands

I have read this submission. I believe that I have an appropriate level of expertise to confirm that it is of an acceptable scientific standard.

# Peer review: Referee response for version 2

**DOI:** 10.5256/f1000research.10150.r15805

**Published:** 2016-11-22

**Authors:** Saskia Hiltemann

**Affiliations:** 1 Department of Bioinformatics, Erasmus University Medical Center, Rotterdam, Netherlands

With this revision, the authors have successfully addressed all my previous concerns.

 The installation instructions were much improved, and I was able to set up MetaGenSense without any major difficulty. The availability of a Virtual Machine was especially convenient for quick testing/assessing of the application, and I was able to run the example pipeline on the provided test data easily.

 The manuscript itself was also much improved and I am now happy to approve this manuscript. I think this is a nice example of how one can integrate a LIMS/project management system with Galaxy at the back-end for analysis.

 Minor (optional) remarks:

1. Unless there is a compelling reason not to, please consider adding the requirements.txt file to the github repo, it was a little bit confusing that I had to create this file myself. 
2. Your installation instructions refer to the command "manage.py migrate" but this appears not to be implemented until Django 1.7, yet your documentation lists the requirement "Django==1.6.2". Consider changing the requirement version for Django from 1.6.2 to 1.7 (I had no problem running the rest of the setup using Django 1.7) or changing this part of the installation instructions to fit with Django 1.6.2.
3. Capitalize "galaxy" in last paragraph of the manuscript ( [..]  the tools needed in our metagenomic example workflow, a galaxy instance containing those tools [..])
4. For the VM, it would be useful if you also provided the login credentials for the admin user (mgs_admin) for MGS/Galaxy in your README on github, for those readers who may be interested in seeing how the admin side of the application works.

I have read this submission. I believe that I have an appropriate level of expertise to confirm that it is of an acceptable scientific standard.

# Peer review: Referee response for version 2

**DOI:** 10.5256/f1000research.10150.r15804

**Published:** 2016-09-14

**Authors:** Manuel Corpas

**Affiliations:** 1 Future Business Centre, Cambridge, UK

I appreciate that there have been noticeable improvements, hence I am now able to accept this article with reservations. I hope authors find my suggestions useful.

 I would have preferred if the response letter was more specific, quoting the suggestions offered by the reviewers and directly underneath the author responses showing how they have addressed them. Currently I have no easy way to check how you have addressed my previous comments. The response letter has some grammatical errors. I paste below the bits that are grammatically incorrect. I would recommend that authors have proof read their submitted materials before sending them.

- "according to reviewers remarks” 
- “this application is more design”
- “Description of the software is also a more precise"
- I am unable to run the image http://webext.pasteur.fr/metagensense/metagensense.ova as I do not have the ability to create virtual machines. Perhaps having a set of images that describe exactly the point authors want to make regarding this virtual machine could help the review process. This seems to be done in Figure 2, although the resolution is not sufficient.
- I am unable to read the font inside of Figure 2’s window screenshots. I would like to be able to see what each of them shows. This way I would be able to see how the interface works for each of the stages (e.g., Connection, Project creation, etc.).
- I went to GitHub and I went to "http://metagensense.test.fr:8000” but the answer I got from the server was "metagensense.test.fr refused to connect”.
- In the installation notes of the README.md, the first step points to virtualenv, virtualenvwrapper. I would have appreciated if authors could provide in there how I can install virtualenv. It is just need to take a few instructions from https://virtualenv.pypa.io/en/stable/installation/.

I have read this submission. I believe that I have an appropriate level of expertise to confirm that it is of an acceptable scientific standard, however I have significant reservations, as outlined above.

# Response

**Published:** 2016-11-21

**Authors:** Olivia Doppelt-Azeroual

**Affiliations:** Institut Pasteur, France

Dear Dr. Corpas,

 Thank you for your review and remarks. According to your advice, the documentation, publication and other materials have now been proofread by a colleague.

 The main change since the second version of the publication is the documentation available in the github repository http://metagensense.readthedocs.io/en/latest/?badge=latest. We tried to clarify the following points:

- The documentation on the main github page was simplified and we clearly separated the virtual machine test documentation and the metagensense local installation procedure.
- The documentation available on the platform Read the Docs was proofread and that enabled us to emphasize each step of the installation procedure.  
- We added documentation for the Virtualenv installation procedure
- The Figure 2 which was in a low resolution was updated on the github repository and the publication.

You will find below the answers to your remarks:

 [Q1]

 I am unable to run the image http://webext.pasteur.fr/metagensense/metagensense.ova as I do not have the ability to create virtual machines. Perhaps having a set of images that describe exactly the point authors want to make regarding this virtual machine could help the review process. This seems to be done in Figure 2, although the resolution is not sufficient.

**[A1]**

**The point of the Virtual Machine is to allow users to test the MetaGenSense application before installing it on their local infrastructure.**

**The online documentation available at the URL: http://metagensense.readthedocs.io/en/latest/connection.html now includes a step by step user guide.**

**[**Q2]

 I am unable to read the font inside of Figure 2’s window screenshots. I would like to be able to see what each of them shows. This way I would be able to see how the interface works for each of the stages (e.g., Connection, Project creation, etc.).

**[A2]**

**The image for each stages has been uploaded with a better resolution so the readers can have a better idea of MetaGenSense’s looks without installing the virtual machine. It is available at the URL: https://github.com/pgp-pasteur-fr/MetaGenSense/blob/master/doc/images/metagensense_complete.jpeg**

 [Q3]

 I went to GitHub and I went to "http://metagensense.test.fr:8000” but the answer I got from the server was "metagensense.test.fr refused to connect”.

**[A3]**

**The metagensense.test.fr URL is only available when running the MetaGenSense virtual machine. The documentation available on the github repository has been corrected to clarify this point.**

**[**Q4]

 In the installation notes of the README.md, the first step points to virtualenv, virtualenvwrapper. I would have appreciated if authors could provide in there how I can install virtualenv. It is just need to

 take a few instructions from https://virtualenv.pypa.io/en/stable/installation/.

**[A4]**

**We changed the documentation and added information regarding the installation of a virtual environment.**

 Thank you again for your review. We hope the changes we applied to our github documentation will clarify MetaGenSense's description and its installation procedure.

 Best regards,

 Olivia Doppelt-Azeroual

# Peer review: Referee response for version 1

**DOI:** 10.5256/f1000research.6578.r8902

**Published:** 2015-06-24

**Authors:** Saskia Hiltemann

**Affiliations:** 1 Department of Bioinformatics, Erasmus University Medical Center, Rotterdam, Netherlands

General Comments:

The authors describe their application, MetaGenSense, a web-based application for analysing metagenomic data. It provides a user-friendly interface which combines a LIMS system with a Galaxy backend for computation and workflow management.

The Django framework is very nice, and I think the integration of Galaxy with a LIMS system is very useful and something many readers will be interested in.

However, many aspects of this application are tailored specifically to the authors' local setup. I installed parts of the application, but since no information is provided on how to install the various components (LIMS/Django/KRONA/BioBlend), and since the Galaxy server used in the code was not accessible to me, it was not fully functional, and because documentation was lacking, it was unclear to me how to proceed with the setup.

To make this work more valuable to the readers, the following additions would be helpful:

1. Installation instructions for the code on GitHub. The readme file is empty at the moment.
  How to install the various components (LIMS, Django UI, KRONA, BioBlend)? And how to connect the different components together? How to configure the webserver correctly (apache/nginx/other)? Which parts of the code are specific to the authors' local setup and need to be adapted when readers install their own MetaGenSense instance?
2. A description of the Galaxy workflows used by the authors would also be very interesting, which tools are used? are they available from the Galaxy tool shed? 
3. Either create a demo server with an example project or add screenshots of the application to the manuscript. The UI looks quite nice, show it to the readers.
4. The case study section is very technical, and would be enhanced by showing the use-case in terms of a real biological example, add screenshots of a real-world analysis to the various steps in this section.

Minor Edits:

1. Capitalize the word "Galaxy" throughout. 
2.  In section "Bioinformatics and HTS projects", BiobBlend --> BioBlend

I have read this submission. I believe that I have an appropriate level of expertise to confirm that it is of an acceptable scientific standard, however I have significant reservations, as outlined above.

# Response

**Published:** 2015-08-06

**Authors:** Olivia Doppelt-Azeroual

**Affiliations:** Institut Pasteur, France

Thank you very much for your critical review. We posted the answers for your remarks underneath them in bold. 

 To make this work more valuable to the readers, the following additions would be helpful:

1. Installation instructions for the code on GitHub. The readme file is empty at the moment.
**Yes, the README file is now complete. It contains installation information. Please tell us if you feel that it gathers all useful information to install and configure MetaGenSense.**
****
** Along with the version 2 of MetaGenSense publication, we built a release of the software. It is available at the URL (** **https://github.com/pgp-pasteur-fr/MetaGenSense/releases/tag/v1.0** **) **
  How to install the various components (LIMS, Django UI, KRONA, BioBlend)? And how to connect the different components together? How to configure the webserver correctly (apache/nginx/other)? Which parts of the code are specific to the authors' local setup and need to be adapted when readers install their own MetaGenSense instance?
**Concerning the LIMS, Django, BioBlend, everything is in the application itself so the connection between the components is natively implemented.**
**For the apache, it is directly linked to Django which is deployed on an apache server. It is very well documented on this url:**
**https://docs.djangoproject.com/en/1.8/howto/deployment/ **
**For KRONA, the javascript which enables the taxonomy distribution exploration is actually generated by a tool, installed in Galaxy. At the Institut Pasteur, it is part of an in-house package which gathers several tools for taxonomy analyses. Those tools are available on GitHub now (it was not a year ago) at the URL:** **https://github.com/C3BI-pasteur-fr/taxo_pack** **. To test those tools, we implemented a Virtual Machine image pre-configured to test MetaGenSense directly on a web browser. Instructions are also available in the GitHub README file.**
****
** For your last question, please look at the “set the settings” part of the README file. **
2. A description of the Galaxy workflows used by the authors would also be very interesting, which tools are used? are they available from the Galaxy tool shed?
**The workflow included on the virtual Machine Galaxy instance contains a light version of our metagenomic analysis workflow. A small fastq file is also included to test it. **
3. Either create a demo server with an example project or add screenshots of the application to the manuscript. The UI looks quite nice, show it to the readers.
**Thank you for that. Yes, another reviewer suggested that we put screenshots of the UI. We added a figure that resumes all the windows and their use for each step. The figure is the available at this url:** **https://github.com/pgp-pasteur-fr/MetaGenSense/blob/master/doc/images/metagensense_complete.jpeg** **as well as each of the steps (in bigger picture). This figure is now added to the publication. **
4. The case study section is very technical, and would be enhanced by showing the use-case in terms of a real biological example, add screenshots of a real-world analysis to the various steps in this section.
**Please tell us if the figure resumes well the various steps. **

**For the minor remarks, they are all included in the second version of the publication. Thank you for your critical review of our article.**

# Peer review: Referee response for version 1

**DOI:** 10.5256/f1000research.6578.r8217

**Published:** 2015-06-22

**Authors:** Manuel Corpas, Benedikt Rauscher

**Affiliations:** 1 Future Business Centre, Cambridge, UK; 2 The Genome Analysis Center, Norwich, UK

MetaGenSense is intended to help find pathogen data in metagenomic data created through next generation sequencing. Measured data including the sequencing reads and metadata are fed into a Laboratory Information Management System (LIMS). The application can fetch that information and pipe it into predefined Galaxy workflows, run them and visualise the output via a framework called KRONA.

The introduction to the article is perhaps too long (almost half of the article). There are sections that are not necessarily related to the research presented here, e.g.,  the paragraph focusing on the assembly problem of next generation sequencing reads.  It would be useful, however, that the authors give a more comprehensive introduction into metagenomics as this topic is only covered very briefly at the beginning of the introduction.

The section on the software tool itself is very technical. I have trouble identifying a clear train of thought. Also it could be shorter and more precise. The case study does not really seem to be a case study on how the application can be used to actually find pathogen information in metagenomic data but is more like a step by step protocol on how to use the application. I suggest that this kind of information should be moved to the documentation and that instead a concrete biological example is demonstrated in the article. Moreover, the title says that MetaGenSense can visualise its results. However, this is not shown in the article. Therefore I would advise the authors to consider replacing the current figure with a figure demonstrating the results of a concrete biological use case.

The discussion and conclusion seem to be a summary rather than a discussion.

MetaGenSense seems to lack many of the standard requirements of a quality software product

- We could not find any documentation. The README file on GitHub does not contain any information.
- The last update to the code was months ago, suggesting that the program is not being developed and maintained actively.
- We could not find any tests.
- We could not find any examples, demos or even screenshots of the interface.

Therefore we are not convinced that MetaGenSense adheres to the journal’s quality standards.

We believe that the article should be revisited and documentation, live examples and tests should be added to the software before the article should be indexed.

We have read this submission. We believe that we have an appropriate level of expertise to state that we do not consider it to be of an acceptable scientific standard, for reasons outlined above.

# Response

**Published:** 2015-08-28

**Authors:** Olivia Doppelt-Azeroual

**Affiliations:** Institut Pasteur, France

We tried to answer each of your comments. A second version of the publication will be available soon. 

 Thank you for your critical review of our article.

Reviewer Comment:

 MetaGenSense is intended to help find pathogen data in metagenomic data created through next generation sequencing. Measured data including the sequencing reads and metadata are fed into a Laboratory Information Management System (LIMS). The application can fetch that information and pipe it into predefined Galaxy workflows, run them and visualise the output via a framework called KRONA.

 The introduction to the article is perhaps too long (almost half of the article). There are sections that are not necessarily related to the research presented here, e.g., the paragraph focusing on the assembly problem of next generation sequencing reads.  It would be useful, however, that the authors give a more comprehensive introduction into metagenomics as this topic is only covered very briefly at the beginning of the introduction.

**Introduction is now shorter in the second version of the publication. It was modified in a way that more emphasis is made on the importance of a true metagenomics component for HTS-type analyses. Information regarding assembly problems are indeed of secondary importance, and they were removed for the new version.**

**Reviewer Comment:**

 The section on the software tool itself is very technical. I have trouble identifying a clear train of thought. Also it could be shorter and more precise. The case study does not really seem to be a case study on how the application can be used to actually find pathogen information in metagenomic data but is more like a step by step protocol on how to use the application. I suggest that this kind of information should be moved to the documentation and that instead a concrete biological example is demonstrated in the article. Moreover, the title says that MetaGenSense can visualise its results. However, this is not shown in the article. Therefore I would advise the authors to consider replacing the current figure with a figure demonstrating the results of a concrete biological use case.

**Thank you for this interesting remark; the use-case part of the article was modified to be more precise. Moreover, a user can now use MetaGenSense on a concrete biological dataset. We created a virtual machine image preconfigured to directly use MetaGenSense with a web browser. A fastq file, as well as a light version of our metagenomic Galaxy workflow is included. The user can connect to the framework, launch an analysis, explore the framework, as well as the results.**

**Moreover, we also would like to change the title of the publication for it to be more adapted to our approach; replacing visualisation by the word exploration which is really the goal of an application like MetaGenSense. The title of the second version of the article is:**

**"MetaGenSense : A web application for analysis and exploration of high throughput sequencing metagenomic data."**

**Reviewer Comment:**

 The discussion and conclusion seem to be a summary rather than a discussion.

 MetaGenSense seems to lack many of the standard requirements of a quality software product

- We could not find any documentation. The README file on GitHub does not contain any information.
**MetaGenSense README file is now complete. We also have written on a userGuide available directly on our Github repository through the web tool readthedocs :** **http://metagensense.readthedocs.io** **.**

**Reviewer Comment:**

- The last update to the code was months ago, suggesting that the program is not being developed and maintained actively.
**The code in the GitHub repository was committed just before the submission of the article. A few debug and add-ons have been implemented since the previous release. **

**Reviewer Comment:**

- We could not find any tests.
**As mentioned earlier, we implemented a Virtual Machine Image containing the infrastructure to test our framework. It is pre-configured so that any user can start using MetaGenSense with a web browser. It is available on the Institut Pasteur server as it was too big to be uploaded on GitHub (** http://webext.pasteur.fr/metagensense/metagensense.ova **). Documentation about this image is available on the README file of our GitHub repository. As Metagenomic analyses are time and storage consuming, we made available a very light version of our workflow with a small fastq file. However, it is enough to test the framework and to understand how the database, the Django framework and the related Galaxy instance are working together. **

**Reviewer Comment:**

- We could not find any examples, demos or even screenshots of the interface.
**For the screenshots, we added a picture of the framework at each step of its use. It is also available at the url:** **https://github.com/pgp-pasteur-fr/MetaGenSense/blob/master/doc/images/metagensense_complete.jpeg**
**Moreover, as the screenshots are small, they are also available on our github repository in the doc directory. **

# Peer review: Referee response for version 1

**DOI:** 10.5256/f1000research.6578.r8901

**Published:** 2015-06-19

**Authors:** Peter Li

**Affiliations:** 1 GigaScience, Hong Kong SAR, China

**General comments**

The authors have developed MetaGenSense, a web-based application for analysing metagenomics data. MetaGenSense also acts as a laboratory information management system, managing the metagenomics data and the results of their analysis by Galaxy workflows.

The work done by the authors sounds promising since I am a fan of Galaxy and of the Django framework. However, I believe the paper can be much improved by providing additional details in a number of places.

For example, it would be interesting to learn more about the two prototyped workflows for analysing metagenomics data which are alluded to in the "Pre-designed Galaxy workflow" section on page 5.

The "Case study - use example" section could also be improved by providing screenshots of the MetaGenSense GUI which are relevant to each or some of the steps. At the moment, I have no idea what the GUI for MetaGenSense looks like since there is also no example instance of MetaGenSense available on the Web which would have been useful for reviewing this paper.

In the final paragraph, it is stated that MetaGenSense can be easily deployed but looking at the source code in https://github.com/F1000Research/MetaGenSense, there appears to be a lack of documentation to enable me to do this. For example, which version of Django should I use with MetaGenSense? How do I get the MetaGenSense source code integrated with the Django framework? The LIMS for MetaGenSense uses PostgreSQL; how do I set up this database and get it linked to MetaGenSense? How do I provide MetaGenSense with access to a Galaxy server? I think the authors need to provide this type of information to help readers install MetaGenSense for the authors' source code to be more useful.

**Minor corrections**

Page 3:

Paragraph 4

Pacific Bioscience should be Pacific Biosciences.

Paragraph 7

BiobBlend should be BioBlend.

Page 4

worflow_remote should be workflow_remote

like BWA through galaxy - should be, "like BWA through Galaxy..."

Page 5

Case study - use example: Need to be consistent by starting bullet points with capital letters.

I have read this submission. I believe that I have an appropriate level of expertise to confirm that it is of an acceptable scientific standard, however I have significant reservations, as outlined above.

# Response

**Published:** 2015-08-06

**Authors:** Olivia Doppelt-Azeroual

**Affiliations:** Institut Pasteur, France

Thank you for your review. For each of your remarks, we wrote responses in bold:

 For example, it would be interesting to learn more about the two prototyped workflows for analysing metagenomics data which are alluded to in the "Pre-designed Galaxy workflow" section on page 5.

**This publication aims to present an application combining as you wrote above a lims, a direct link to any Galaxy and a way to sort and manage Galaxy results. The workflow you choose to use is totally arbitrary. In essence, MGS was designed in a way that any Galaxy workflow can be plugged-in.**

****

**To facilitate testing, we implemented a Virtual Machine image pre-configured to test MetaGenSense directly on a web browser. Instructions for download and use are available in the GitHub README file. However, as instructions for installation of the framework are now available, any developer can download and link MetaGenSense to his Galaxy.**

 The "Case study - use example" section could also be improved by providing screenshots of the MetaGenSense GUI which are relevant to each or some of the steps. At the moment, I have no idea what the GUI for MetaGenSense looks like since there is also no example instance of MetaGenSense available on the Web which would have been useful for reviewing this paper.

**Yes we agree with you, we omitted to add screenshots in the first version of the article. We added a new figure that resumes all MetaGenSense steps and functionalities. It is available at:** **https://github.com/pgp-pasteur-fr/MetaGenSense/blob/master/doc/images/metagensense_complete.jpeg** **.** **It is now the 2nd figure of the article. Moreover all the small figures (steps) are also available in the doc directory of our GitHub repository.**

 In the final paragraph, it is stated that MetaGenSense can be easily deployed but looking at the source code in https://github.com/F1000Research/MetaGenSense, there appears to be a lack of documentation to enable me to do this. For example, which version of Django should I use with MetaGenSense? How do I get the MetaGenSense source code integrated with the Django framework? The LIMS for MetaGenSense uses PostgreSQL; how do I set up this database and get it linked to MetaGenSense? How do I provide MetaGenSense with access to a Galaxy server? I think the authors need to provide this type of information to help readers install MetaGenSense for the authors' source code to be more useful.

**We improved the documentation on our github repository (** **https://github.com/pgp-pasteur-fr/MetaGenSense** **) with elements facilitating deployment. We completed a README file containing well-informed installation and configuration instructions. We also wrote a UserGuide working with readTheDocs documentation tool (** **http://metagensense.readthedocs.io** **).  Don’t hesitate to tell us if parts of the documentation are still a bit blurry.**

**We will adapt the manuscript with all your minor corrections and suggestions.**

****

**Thank you very much for your review.**

# Response

**Published:** 2016-09-27

**Authors:** Peter Li

**Affiliations:** GigaScience, Hong Kong

To test the authors' software, it will take over 6 hours for me to download the 2.7 GB machine image provided by the authors. I think this is due to my office internet connection being very slow and because of the fact that I am based in the Far East. Unfortunately, waiting 6 hours plus for the machine image to download is not really practical for me but this is not a fault of the authors.

 I had a look at the instructions to install MetaGenSense which are available from the README.md file in their GitHub repository in an attempt to manually install the software. I feel that the instructions are minimal, for example, the installation requires a requirements.txt file but I could not find one in the MetaGenSense repo. You would need to know that the contents of requirements.txt are listed at the start of the manual installation instructions. The installation instructions also rely on the user doing background reading to find out how to install and use MetaGenSense's dependencies, for example, virtualenv and a database server.

# Response

**Published:** 2016-11-21

**Authors:** Olivia Doppelt-Azeroual

**Affiliations:** Institut Pasteur, France

Dear Peter Li,

 Thank you for your review and remarks.

 Since last August, we asked a non familiar with MetaGenSense colleague to read and install locally the application. This exercise has enabled us to clarify some points. We simplified the README file (https://github.com/pgp-pasteur-fr/MetaGenSense) and added significant details to the installation procedure part in the readthedocs documentation:

- As you mentioned, the virtualenv installation procedure has been added.
- We also clarified the part concerning the requirements, i.e. the developer needs to copy the three requirements in a requirements.txt file and use the “pip” command to install them. http://metagensense.readthedocs.io/en/latest/installation.html#requirements
- The part concerning the database configuration has also been a bit modified in order to be clearer.

We hope the changes applied recently enable you to successfully install MetaGenSense.

 Best regards,

 Olivia Doppelt-Azeroual
